# Dose-Dependent Thresholds of 10-ns Electric Pulse Induced Plasma Membrane Disruption and Cytotoxicity in Multiple Cell Lines

**DOI:** 10.1371/journal.pone.0015642

**Published:** 2011-01-26

**Authors:** Bennett L. Ibey, Caleb C. Roth, Andrei G. Pakhomov, Joshua A. Bernhard, Gerald J. Wilmink, Olga N. Pakhomova

**Affiliations:** 1 711th Human Performance Wing, Radio Frequency Radiation Branch, Air Force Research Laboratory, Brooks City-Base, San Antonio, Texas, United States of America; 2 Frank Reidy Research Center for Bioelectrics, Old Dominion University, Norfolk, Virginia, United States of America; 3 General Dynamics Information Technology, Brooks City-Base, San Antonio, Texas, United States of America; University of California at Berkeley, United States

## Abstract

In this study, we determined the LD_50_ (50% lethal dose) for cell death, and the ED_50_ (50% of cell population staining positive) for propidium (Pr) iodide uptake, and phosphatidylserine (PS) externalization for several commonly studied cell lines (HeLa, Jurkat, U937, CHO-K1, and GH3) exposed to 10-ns electric pulses (EP). We found that the LD_50_ varied substantially across the cell lines studied, increasing from 51 J/g for Jurkat to 1861 J/g for HeLa. PS externalized at doses equal or lower than that required for death in all cell lines ranging from 51 J/g in Jurkat, to 199 J/g in CHO-K1. Pr uptake occurred at doses lower than required for death in three of the cell lines: 656 J/g for CHO-K1, 634 J/g for HeLa, and 142 J/g for GH3. Both Jurkat and U937 had a LD_50_ lower than the ED_50_ for Pr uptake at 780 J/g and 1274 J/g, respectively. The mechanism responsible for these differences was explored by evaluating cell size, calcium concentration in the exposure medium, and effect of trypsin treatment prior to exposure. None of the studied parameters correlated with the observed results suggesting that cellular susceptibility to injury and death by 10-ns EP was largely determined by cell physiology. In contrast to previous studies, our findings suggest that permeabilization of internal membranes may not necessarily be responsible for cell death by 10-ns EP. Additionally, a mixture of Jurkat and HeLa cells was exposed to 10-ns EP at a dose of 280 J/g. Death was observed only in Jurkat cells suggesting that 10-ns EP may selectively kill cells within a heterogeneous tissue.

## Introduction

Short-duration, high voltage electric pulses (micro and millisecond duration) cause defects in the plasma membrane of cells [1], [2], [3]. These membrane defects can allow the transient passage of impermeable molecules by direct diffusion through aqueous pores or by electrophoresis. This technique has been used in conjunction with toxic agents (e.g. bleomycin) to kill specific cells and tissue, a technique called electrochemotherapy [4], [5], [6], [7]. Electric pulses can also cause irreversible membrane breakdown resulting in cell death, termed irreversible electroporation (IE) [8], [9], [10], [11], [12], [13]. Killing of unwanted cells and tissue by IE and electrochemotherapy has shown to be effective in the treatment of cancers [4], [6], [9], [11], [12], [14]. The use of ultrashort electric pulses (USEP) has emerged as a novel modality to kill cells based on theoretical and empirical results showing that USEP can cause intracellular membrane poration. Intracellular poration has been hypothesized to lead to apoptotic cell death resulting in an ordered removal of tissue by the body [15], [16], [17], [18], [19], [20]. *In vivo* experiments have also shown USEP to be a successful treatment for tumors [21], [22], [23]. USEP, because of their innate high frequency components, also have the potential of being delivered remotely by close-range antenna highlighting the importance of studying USEP-induced bioeffects [24], [25], [26], [27].

Despite theoretical predictions of intracellular poration and empirical results, it remains unclear whether the plasma membrane plays a role in triggering cell death following USEP exposure. Therefore, the aim of this research was to measure cell survival in relation to disruptions in the plasma membrane across several commonly studied cell lines. Previous studies have shown that different cell lines (HeLa, Jurkat, U937, HL-60, etc.) require different intensities of USEP exposures to cause death [28], [29], [30]. Unfortunately, the exposure parameters (pulse amplitude, duration, repetition rate, and number) and the cellular environment (exposure buffer) varied considerably across these studies resulting in an unclear understanding of the USEP exposure requirements for cellular death across multiple cell lines. However, it is believed that morphological and physiological differences between various cell types do influence susceptibility to injury and death by ultrashort electrical stimuli [28]. In previous work, we demonstrated that the dose required to kill Jurkat cells is substantially less than that required to kill U937 cells when exposed to 10-ns EP [29], [31]. In addition, we showed that this dose-dependent susceptibility appears only when the pulse duration is short (<300 ns) [29].

Recent work utilizing patch clamp and fluorescent microscopy has provided sufficient evidence that the plasma membrane is not spared by USEP [29], [32], [33], [34], [35], [36], [37]. Whole-cell conductance measurements in multiple cell types (CHO-K1, GH3, Jurkat, and HeLa) have shown significant changes at low exposure levels suggesting formation of long-lasting nanopores (minutes) in the plasma membrane. Results have shown a dose-dependence of nanopore formation for single and multiple pulse exposures at multiple pulse widths [36]. In agreement with patch clamp results, previous groups studying death caused by USEP have observed fast externalization (within minutes) of PS residues without uptake of propidium (Pr) iodide suggesting changes in membrane organization without large pore formation [20], [29], [34], [38], [39]. This work shows that USEP can have profound effect on the plasma membrane and that internal membrane permeabilization unlikely exists independently. A clear connection between effects on the plasma membrane of cells exposed to ultrashort pulses and cell death remains unproven. Without such a connection it will be impossible to properly guide future work aimed at determining the mechanism(s) that cells utilize to repair their membranes or die following USEP exposure and whether it is truly mechanistically different than death caused by longer duration pulses.

## Materials and Methods

### Cell Lines and Propagation

Experiments were performed in five cell lines, Jurkat clone E6-1 (human T-lymphocytes), U-937 (human monocytes), GH3 (rat pituitary), CHO-K1 (hamster ovarian epithelial), and HeLa (human cervical epithelial). The cells were obtained from ATCC (Manassas, VA) and propagated at 37°C with 5% CO_2_ at 95% humidity in air. Different media were used for culturing each cell type as per ATCC guidelines. The media and its components were purchased from ATCC and supplemented with 1% penicillin/streptomycin (ATCC). It was critical that the cell lines be exposed to 10-ns EP in a comparable way requiring the temporary suspension of adherent cultures; HeLa, GH3, and CHO-K1 cells were harvested during the logarithmic growth phase by rinsing the cells in 0.25% trypsin (ATCC) for up to 5 minutes, pelleted by centrifugation, counted on a Z1 particle counter (Beckman Coultier, Miami, FL), and resuspended at 1200 cells/µL in their respective growth medium. Jurkat and U937 cells were harvested during the logarithmic growth phase, pelleted by centrifugation, counted on the Z1 particle counter, and resuspended at 1200 cells/µL in their respective growth medium.

### Exposure to 10-ns Pulses

The 10-ns exposure system has been described previously [29], [31], [40]. In brief, to produce a 10-ns EP, a Blumlein line circuit was charged from a high-voltage DC power supply until a breakdown voltage was reached across a spark gap in a pressurized switch chamber. The breakdown voltage (and, consequently, the voltage of USEP delivered to the sample) was varied from 15 to 40 kV by changing the pressure of SF_6_ gas in the switch chamber. The pulser control system included a programmable gas regulator, pulse counter, and GPIB outputs for communication with the high voltage power supply and digital high-speed oscilloscope (TDS3052B, Tektronix, Wilsonville, OR). The control system communicated with a PC using a specialized program written in LabVIEW® (National Instruments, Austin, TX).

For exposure, cells suspended in complete growth medium were dispensed into conventional electroporation cuvettes with 1-mm (150-µL volume) or 2-mm (400-µL volume) gap between the electrodes (BioSmith Biotech, San Diego, CA). The amplitude, number of pulses, and pulse shape were recorded for every exposure using a custom built pulsing controller and oscilloscope. The electroporation cuvettes were exposed to USEP at a room temperature (21–23°C). In each series of experiments, different EP treatments, including sham exposure, were alternated in a random sequence. Once filled with the cell suspension, cuvettes were subjected to USEP treatment within 20 minutes. All exposures were carried out at a pulse repetition frequency range of 1.7–2.2 Hz.

### Dosimetry

Table 1 displays the exposure parameters used in the experimentation and the calculated dose delivered to the cuvette. Due to the variability in the pulse amplitude generated from the spark gap, electric fields were measured for every pulse and the average value was used to calculate the dose [31]. In this table, we show the number of pulses (10, 30, 100, 300, 1000) and average electric field amplitudes (65, 105, 150, 285 kV/cm) used in the exposures. The resultant dose was calculated as in previous publications [29], [31]. The individual column on the right shows the average dose as calculated by combining doses of similar magnitude into a single data point. This averaging was performed to simplify the presentation of cell survival data on the logarithmic scale.

**Table 1:** Exposure parameters used in this study and the calculated dose delivered to the cuvette.

Amplitude	65 kV/cm	105 kV/cm	150 kV/cm	285 kV/cm	Average Dose (J/g)
Pulse Number					12
10	5	14	28	101	40
30	16	41	84	302	120
100	52	137	279	1007	379
300	157	410	837	3022	1070
1000	524	1367	2790	10072	2906
					10072

The exposure parameters are shown in the top row and left column of the table. The dose calculated from these exposures is presented in corresponding cells. The average dose, generated by combining doses at a similar magnitude, is presented in the right-most column.

### Cell Survival

To obtain survival data for the 5 different cell cultures at 24 hours post exposure, an MTT Cell Proliferation Assay (3-(4,5-Dimethylthiazol-2-yl)-2,5-diphenyltetrazolium bromide, ATCC, Manassas, VA) was used. Exposed cells were aseptically aliquoted into a 96-well plate, in triplicates at 50×10^3^ cells/well, and diluted to 100 µl with fresh growth medium. The plate was incubated at 37°C, with 5% CO_2_ in air. At 22 hours after EP treatment, 10 µl of MTT reagent was added to each well, and incubation continued for 2 hours. Formed blue formazan crystals were dissolved by adding the solubilization buffer (100 µL/well) and placing the plate on an orbital shaker overnight. Absorbance at 570 nm was read the next day using a Synergy HT microplate reader (BioTEK, Winooski, VT), and the readings in EP-exposed samples were normalized to parallel controls.

### Flow Cytometry and Confocal Microscopy

Upon exposure to 10-ns EP, aliquots of the cellular suspension were added to a tube containing full medium, 0.1% Annexin V-FITC (BD Pharmingen, San Diego, CA), and 0.02% propidium iodide (Sigma-Aldrich, St. Louis, MO). The cells were incubated for 15 minutes at room temperature, in amber tubes, in the presence of the dyes to allow for uptake of Pr and adequate binding of Annexin V-FITC [18], [41], [42]. Following this incubation period, the cells were quickly resuspended by mild vortexing and analyzed with an Accuri C6 Flow Cytometer (Accuri Cytometers, Inc., Ann Arbor, MI). Samples were run in triplicate at a set analysis volume (75 µL). Sham-exposed samples and those treated with 0.005% digitonin were used as negative and positive controls, respectively. Flow analysis was performed by gating the cellular population and appropriately compensating the fluorescent overlap between Annexin V-FITC and Pr channels. Percentage of positive fluorescent expression in the gated population was measured by applying a threshold to the sham population, allowing for approximately 5–10% of the cells to appear as positive. This binary analysis method was used to ensure highest possible sensitivity for membrane permeabilization. The expression of PS was measured following exposure of each cell type to 100, 10-ns pulses at four distinct E-field intensities (35, 60, 105, 150 kV/cm). The uptake of Pr was measured by exposing each cell population to a high E-field (200 kV/cm for Jurkat, GH3 and 285 kV/cm for U937, HeLa, CHO-K1) at increasing pulse numbers (10, 30, 100, 300). Survival data was processed and plotted using Grapher® software (Golden Software, Golden, Colorado). Flow cytometry results were processed in C6 software (Accuri Cytometers, Inc., Ann Arbor, MI) and FCSExpress software (DeNovo Software, Los Angeles, CA). Final analysis and presentation of flow cytometry results were also generated using Grapher®.

Confocal images of cells were taken following USEP exposure to validate flow cytometry measurements. Cells exposed to either 100 pulses at 105 kV/cm, or 0.005% digitonin, or sham-exposed, were stained similarly to the above protocol for flow cytometry. Following the labeling procedures, cells were placed into a dish containing a glass bottom coverslip (MatTek Corp, Ashland, MA) and placed on a Zeiss 710 LSM microscope (Zeiss MicroImaging, Thornwood, NY). Fluorescent images of Jurkat were captured simultaneously by two PMT's, set for Pr (>590 nm) and FITC (500–550 nm) emission wavelength ranges, using 488 nm argon laser excitation through a 20X, 0.8 NA objective. Brightfield images were also captured using the 488 nm argon laser and the transmission PMT channel. Images were processed using Zen® software (Zeiss MicroImaging, Thornwood, NY).

### Calcium Measurement and Calcium-doped Exposure

Calcium concentration of each complete medium was measured using a QuantiChrom™ Calcium Assay Kit (BioAssay Systems, Hayward, CA). The kit was used according to the manufacturer's protocol and the samples were read on the BioTek Synergy HT (BioTek, Winooski, VT) at 612 nm. Using a standard curve of known calcium dilutions, the concentration of calcium for each cell line's complete medium was determined. To investigate the impact of additional calcium within the exposure medium, calcium chloride was added to Jurkat media to final calcium concentrations of 2.1 mM or 5.1 mM. Jurkat cells were placed in the calcium-doped medium and exposed to increasing pulse numbers at 60 kV/cm. Following exposure the MTT assay was run as previously described.

### Effect of Trypsin on Cell Survival

In order to expose adherent cells within an electroporation cuvette, trypsin treatment was unavoidable. To determine whether trypsin treatment has an impact on cell survival, Jurkat cells were exposed to trypsin to mimic an equivalent experimental protocol as employed for the adherent cell lines. To do this, Jurkat cells were rinsed in a PBS (ATCC) solution and suspended in a 5% trypsin solution. After 5 minutes, cell medium was added to the flask and the cells were allowed to rest for 30 minutes. Cells were exposed to 60 k V/cm using 10, 30, 100, or 300 pulses. Following exposure, the cells were placed in a well plate and allowed to recover over a 24-hour period. MTT assay was used to assess cellular survival.

### Simultaneous Exposure of Jurkat and HeLa Cell Lines

It was unclear whether the sensitivity observed in single cell exposures would hold true in a heterogeneous sample. To study this, HeLa and Jurkat cells were counted using a Z1 particle counter (Beckman Coultier, Miami, FL) and mixed at a 50% cell ratio in complete growth medium (RPMI 1640 media with 10% FBS and 1% pen/strep). The heterogeneous mixture was allowed to grow for 96 hours to eliminate any artifacts brought on by sudden change of media for HeLa cells. Prior to exposure, the supernatant containing Jurkat cells was removed and trypsin was used to isolate HeLa cells. The two populations were then recounted and remixed within an electroporation cuvette at 50% cell ratio for exposure. Cells were exposed to 100 pulses at 0 and 150 kV/cm. The exposed populations were plated and allowed to grow for 24 hours in RPMI 1640 medium. Following that growth period Jurkat cells were removed with the supernatant and separate MTT assays were run for both cell lines.

## Results and Discussion

### Cell Viability

**Figure 1:**
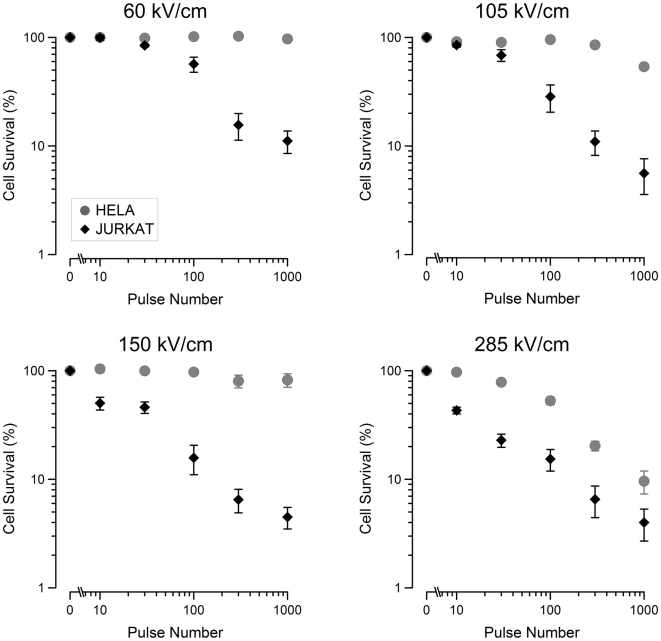
Jurkat and HeLa survival decreases with increasing electric field amplitude and pulse number. Cell survival curves for Jurkat and HeLa cells exposed to 60, 105, 150, and 285 kV/cm. The data shows that the number of cells that survive post-exposure decreases with increasing electric field and pulse number. For all exposures, Jurkat cells are more sensitive than HeLa cells. Data points represent the average survival. (mean +/−s.e., n = 3–5).

Figure 1 shows the cell survival recorded by MTT for Jurkat and HeLa cells exposed to increasing pulse numbers at 60, 105, 150, and 285 kV/cm. Jurkat cells appear to be more sensitive to the effects of 10-ns pulse exposure than HeLa cells at all exposure levels. Increased death with increasing pulse number or pulse amplitude is seen in both cell lines. As in the previous work, a resistive tail remains within the populations; this is due either to non-uniform sample exposure or to a subpopulation of cells that are abnormally resistant [29]. In contrast to Jurkat cells, HeLa cells respond only to the highest electric field and show an increasing effect with increasing pulse number.

**Figure 2:**
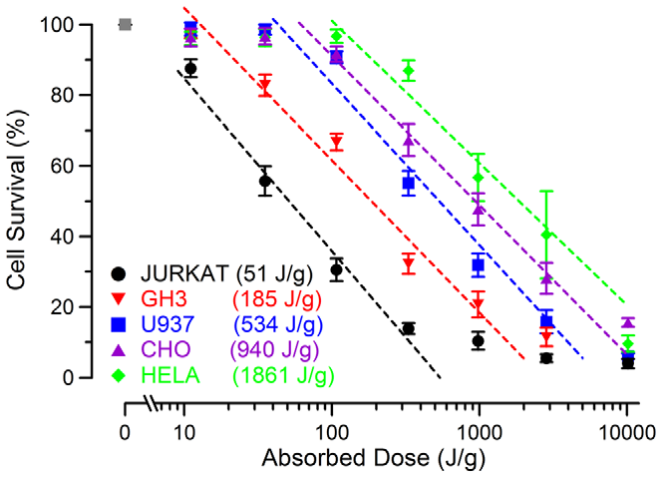
Cell survival as determined by dose in several cell types. Mean survival values (+/− s.e., in % relative to sham-exposed control, n = 3–5) are plotted against the dose delivered to the cuvette. Logarithmic fit lines (dashed) reveal significant differences between cell populations. LD_50_ values (J/g) are displayed in the legend for each population as calculated using the logarithmic fit.

In Figure 2, we show the resulting cell survival data for all 5 cell types as related to the average dose calculated in Table 1. A logarithmic fit was applied to the data to calculate the point at which 50% of the cells die (LD_50_). HeLa, the most resistant cell line tested, had an LD_50_ of 1861 J/g whereas Jurkat cells, the most sensitive cell line, had an LD_50_ of 51 J/g. This represents a nearly 40 times increase in dose needed to kill one cell type versus another. The slopes of the dose response curves for each cell line are quite similar suggesting that the mechanism responsible for cell death may be the same. Shifts in the dose response curve suggest that acute membrane and internal cellular damage may depend on cellular physiology and/or different cell lines may be better able to repair damage by active and passive mechanisms following 10-ns EP exposure.

### Measuring Plasma Membrane Disruption by Flow Cytometry

To better understand the mechanism responsible for cellular death across the different cell lines, we chose to focus on the plasma membrane. It is unclear whether subtle membrane disruption is, in itself, a stressful enough event to cause cell death. To investigate subtle changes induced in the plasma membrane, we chose to monitor the externalization of phosphatidylserine (PS) molecules on the outer leaflet of the plasma membrane by utilizing FITC-labeled Annexin V dye. The chain of events leading to the externalization of PS following USEP exposure remains unknown with hypotheses proposing lateral diffusion of PS through nanopores within the membrane [34] and calcium influx-induced activation of scramblase [43]. Despite the ambiguity of the mechanism ultimately causing externalization of PS, it remains a reliable marker of membrane disruption known to occur at thresholds well below that for propidium ion uptake.

**Figure 3:**
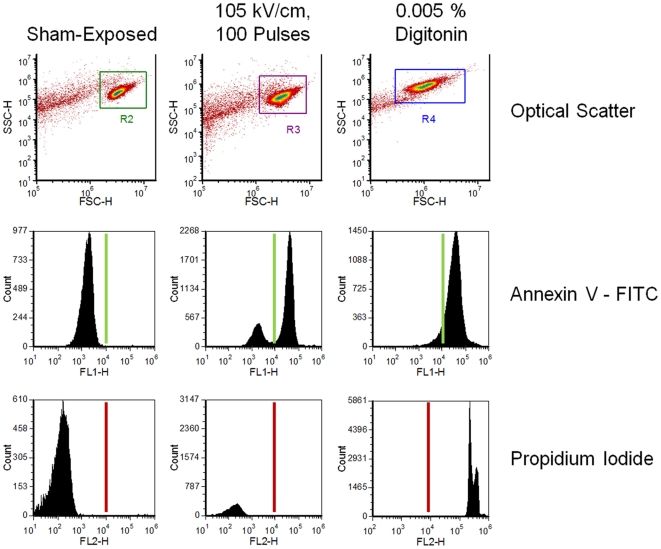
Binary determination of positive phosphatidylserine (PS) expression and propidium uptake by flow cytometry. Plots of the forward and side scatter (top) of sham-exposed cells, and cells exposed to either 105 kV/cm USEP or 0.005% digitonin (positive control for both dyes). Histograms of fluorescent count for Annexin V-FITC (middle) and PI (bottom) are also shown. The solid line represents the threshold used to determine the percent of the cellular population that stains positive for each dye.

**Figure 4:**
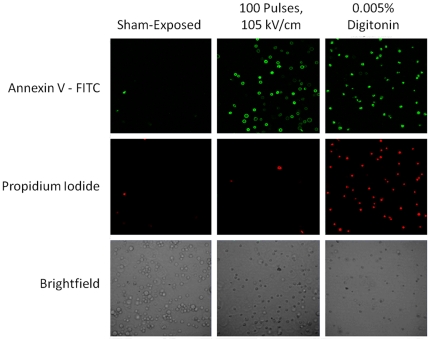
Confocal fluorescent images of Annexin V-FITC expression in USEP-exposed Jurkat. Images detail the fluorescent staining of Jurkat cells following sham-exposure, exposure to 100 USEP at 105 kV/cm, and 0.005% digitonin.

Figure 3 shows raw flow cytometry data from Jurkat cells. The forward and side scatter density plots of the pure cell population along with the compensated reading from both fluorescent channels is shown (for cells exposed to 105 kV/cm, 100 pulses, 0.005% digitonin, or sham-exposed). The USEP-exposed cells show a substantial portion of the population expressing PS on the plasma membrane surface, while remaining impermeable to Pr. In contrast, digitonin causes plasma membrane degradation allowing the influx of Pr and positive staining for PS. Flow cytometry data were obtained for both fluorescent dyes using a threshold based on the sham-exposed population. Figure 4 shows laser scanning confocal microscopy images for each fluorescent dye and a corresponding brightfield image. The first column of images shows minimal positive expression of Annexin V-FITC and Pr occurs in the sham population. The second column of images show that cells exposed to 100, 10-ns EPs at 105 kV/cm positively express Annexin V-FITC with minimal Pr fluorescence. In the third column, the positive control, 0.005% digitonin, shows positive expression of both dyes. While these images can only show a small subset of the exposed population, they agree well with flow cytometry results presented in Figure 3.

**Figure 5:**
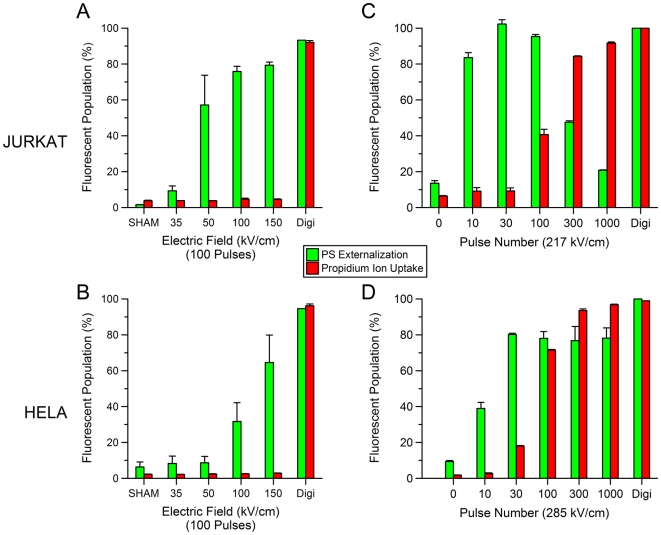
The effect of the E-field and pulse number on USEP-induced externalization of phosphatidylserine (PS) and uptake of propidium ions (Pr). PS and Pr fluorescent expression for Jurkat (A,C) and HeLa (B,D) exposed to increasing electric fields between 0–150 kV/cm at 100 pulses per exposure and 0.005% digitonin. Externalization of PS appears in Jurkat at lower field amplitudes than in HeLa. Both cell lines exposed to digitonin stained positive for both PS and Pr. C and D show percent of Pr positive Jurkat and HeLa after exposure to increasing number of pulses at 217 and 285 kV/cm, respectively. (mean +/− s.d., n = 3 measurements of 25,000 cells).

**Figure 6:**
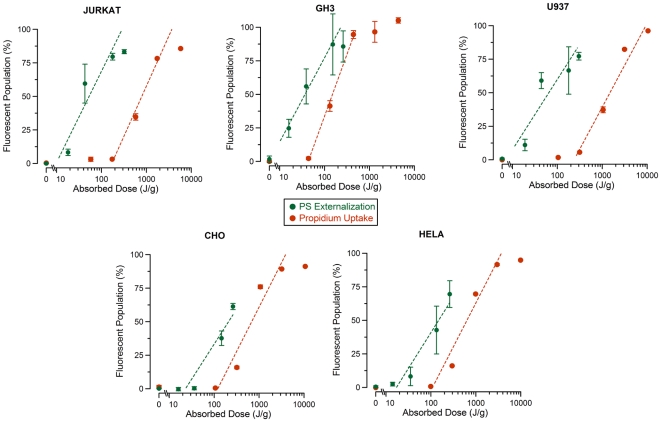
Dose dependence of PS externalization and PI uptake for different cell lines. The percent of the cellular population expressing Annexin V -FITC and Pr was plotted versus absorbed dose for all cell lines tested. Dashed lines represent the logarithmic fit to the data. (mean +/− s.d., n = 3 measurements of 25,000 cells).

Figure 5 shows the flow cytometry results for Jurkat (A,C) and HeLa (B,D) cell lines. The USEP-exposed Jurkat (A) were positive for PS externalization without substantial uptake of Pr, thus agreeing with previous publications [26], [39]. When compared to HeLa (B), Jurkat appear to externalize PS at lower field strength, suggesting that physiological differences between the cells likely cause differences in sensitivity. This finding reinforces previous results obtained using patch clamp technique that showed that HeLa required substantially higher electric field than Jurkat to obtain the same measured changes in plasma membrane conductance [37], [44]. Figure 5C & D show two graphs depicting the results obtained by increasing the electric field to 215 kV/cm for Jurkat and 282 kV/cm for HeLa and increasing the pulse number. In both cell lines, Pr can be brought into the cell if enough pulses are delivered. A substantial drop in cells showing PS externalization is seen at the highest pulse numbers for Jurkat cells. The reason for this drop is unknown, but substantial changes in cell morphology are seen in the forward and side scatter channels at these exposure levels (data not shown). Figure 6 shows the resulting dose-response curves for PS externalization and Pr uptake of all cell lines studied. These data show that the thresholds for PS externalization is lower than for Pr uptake for all cell types tested. Interestingly, the thresholds for PS appear to vary, whereas the thresholds for Pr uptake, with the exception of GH3, appear quite similar.

### Plasma membrane disruption versus cell death

**Figure 7:**
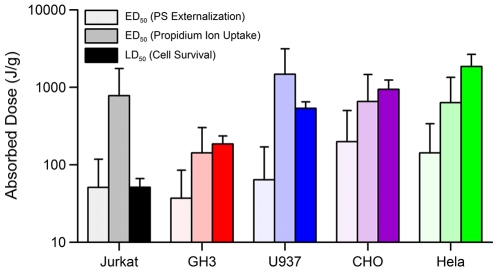
Comparison of ED_50_ for PS externalization and propidium uptake with LD_50_ in different cell lines. Error bars represent the 95% confidence intervals, as calculated from best fit using logarithmic function (see Fig. 2 and 6).

In Figure 7, we have compared LD_50_ to the effective doses (ED_50_) to cause 50% of cells to externalize PS and to cause 50% of cells to uptake Pr. HeLa, GH3 and CHO-K1 become permeable to Pr at doses that are at or below that required for cell death. This result suggests that intracellular membrane permeabilization is not likely to be solely responsible for cell death at 10-ns duration exposures. We conclude that the mechanism by which these cells die may be quite similar to that of irreversible electroporation. The data also shows that Jurkat and U937 have LD_50_'s below that of the ED_50_ for Pr uptake. This result suggests that these cells either experience much delayed Pr uptake (greater than 15 minutes post exposure) and die by a similar mechanism as the other cell lines or die by a completely different mechanism possibly related to regulation of ion imbalance. However, we show that for all cell lines, the dose required for death always exceeds that for PS externalization. These results do in fact show, as seen in Figure 3, that the dose delivered by changing the amplitude or number of 10-ns pulses will have a different effect on different cell types. In other words, these data suggest that killing of cells by 10-ns pulses is potentially selective due to inherent differences in cellular physiology.

### Role of Cell Size in Cell Survival

**Figure 8:**
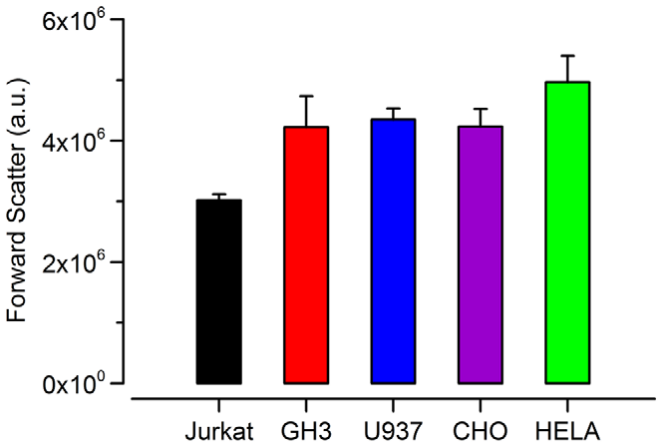
Cell size in tested cell lines as estimated by forward light scattering. Bar height represents the mean of three samples of each cell line and the error bars represent the standard deviation.

Conventional electroporation theory states the radius of the cell in a uniform field increases the induced steady-state transmembrane potential. This theory suggests that a smaller cell will be less apt to form pores than a larger cell due to a smaller induced transmembrane potential [2], [3], [45], [46], [47], [48]. While the assumptions built into this theory apply specifically to micro and millisecond duration pulses, cell size will likely impact the degree of membrane poration for USEP exposures as well [49], [50]. To investigate this, we measured cell size across the studied cell lines using the forward scattering channel of the flow cytometer. Figure 8 shows that Jurkat are the smallest followed by a nearly even sizing of GH3, CHO-K1, and U937, with HeLa being the largest. This result would suggest that Jurkat should be less vulnerable to poration at any given dose than HeLa. Our flow cytometry data suggests the opposite, with the ED_50_ for PS externalization being lower for Jurkat than HeLa. Interestingly, Jurkat have nearly the same ED_50_ for Pr uptake as HeLa, but a large difference is seen in cell survival. This mirrors results by Cemazar *et al.* that showed multiple cell lines exposed to 100 µs EPs, at 1 Hz, have nearly identical thresholds for Pr uptake, but saw large differences in survival. They too noted that the smallest cells (SA-1 sarcoma) proved the most electrosensitive, while ETA, the largest cells proved the least electrosensitive [51]. Agarwal *et al.* studied the response of single cells to ms duration EP and determined that larger cells were easier to permeabilize, but harder to kill as the permeabilization affected only a small portion of the overall cell surface [48]. This theory would hold true for Jurkat and HeLa cell lines given the data presented within this manuscript, but the data for the remaining three cell lines of nearly equal size requires additional explanation. Overall, it does not appear that cell size is the only factor dictating the degree of membrane disruption and cell survival.

### Impact of Extracellular Calcium Concentration on Cellular Survival

**Figure 9:**
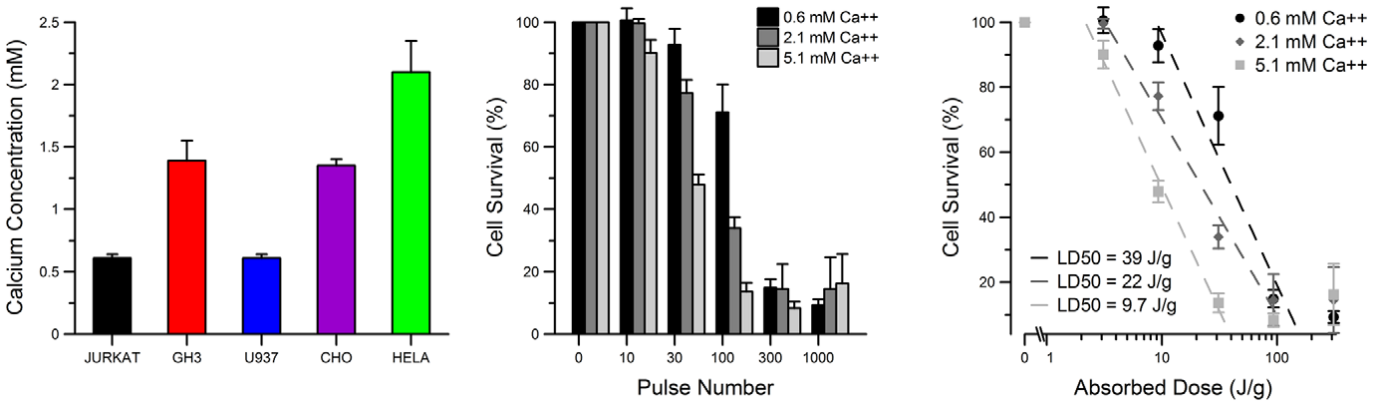
Differences in calcium concentration between different culture media are not responsible for differences in USEP-induced cell death. A: measured calcium concentrations in cell media. B: normalized 24-hr survival of Jurkat in media with modified Ca content. Increasing the external calcium has a negative effect on cell survival. C: a logarithmic fit to data by dose and the predicted LD_50_ values. (mean +/− s.e, n = 3).

The culture media used for each cell type contained different amounts of calcium. The impact of calcium concentration on cell death caused by 10-ns EP is not known and may have contributed to the observed cell-type specific differences in survival. Figure 9A shows the composition of calcium within each individual cell medium measured experimentally. Specifically, Jurkat and U937 are in RPMI 1640 medium, CHO-K1 and GH3 are in F12K medium (GH3 media has 2.5% horse serum), and HeLa is in EMEM medium. Although the differences in Ca concentration between the tested media were small, we sought to better understand the impact of calcium on cell survival. To do this, we exposed Jurkat to USEP in media containing 0.6, 2.1, and 5.1 mM calcium (Figure 9B). These experiments established that, additional calcium has a deleterious effect on the Jurkat cell viability (Figure 9C). Additional calcium causes a distinct left shift of the dose-response curve with the predicted LD_50_ dropping from 39 to 9.7 J/g. Assuming this calcium-dependent increase in cell death is not cell-type specific, one would expect HeLa to have a lower LD_50_ than Jurkat; however the opposite is presented in Figure 2. Furthermore, one could expect that if HeLa were placed in medium containing less calcium, they may be even more resistant to USEP-induced death. This data suggests that, while increasing medium calcium concentration lowers the LD_50_ in Jurkat exposures, the differences in cell survival across the cell lines are unlikely due to inherent differences in medium calcium concentration.

### Impact of Trypsin Treatment on Cell Survival

**Figure 10:**
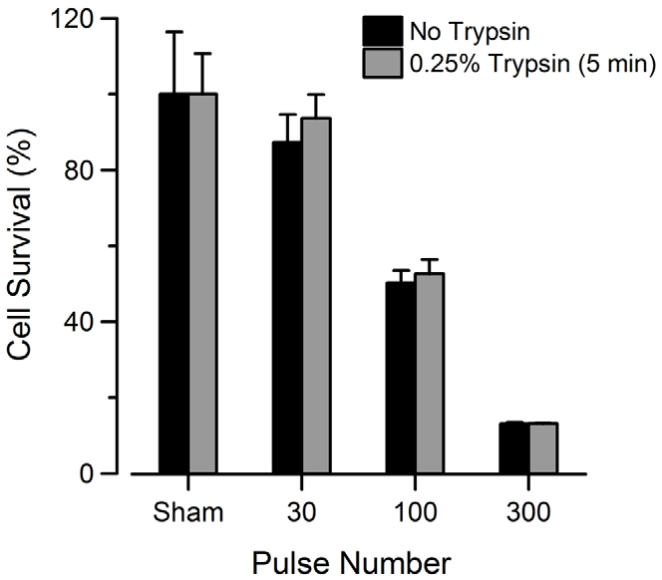
Trypsin treatment does not affect survival of USEP-exposed Jurkat cells. Jurkat cells were exposed to 0, 30, 100, or 300, 10-ns pulses at 60 kV/cm. MTT absorbance measurements were taken 24 hours post exposure. Trypsin treatment appears to have no effect on long-term survival of Jurkat cells. (mean +/− s.e, n = 3).

The use of trypsin to detach adherent cultures for exposure could be a confounding factor affecting the response of adherent cells to USEP. Previous work has shown that cells exposed to trypsin showed trypan blue uptake for up to 90 minutes post exposure implying cells were dead [52], [53]. However, electropermeabilization of plasma membranes can be a lasting effect taking hours to fully recover depending on the exposure conditions and cellular environment [54], [55]. We aimed to determine the effect of trypsin on cell survival within our experiments by treating Jurkat with trypsin similarly to adherent cell lines. Figure 10 shows that Jurkat cells exposed to trypsin have nearly the same degree of cell death as those not exposed to it. Based on these finding, we believe that exposure of cells to trypsin does not impact 24-hour survival following USEP exposure.

### Exposure of Heterogeneous Sample

**Figure 11:**
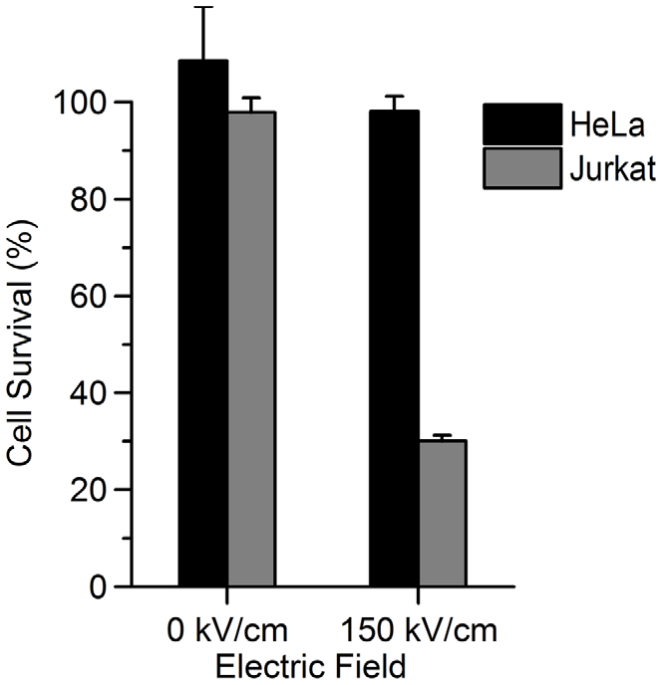
Dose-dependent changes in survival are retained in a heterogeneous cell mixture. Jurkat and HeLa cells were exposed in mixture to a 100 pulse train of 10-ns EP at 150 kV/cm, resulting in efficient killing of Jurkat but little effect on HeLa cells. (mean +/− s.e, n = 3).

Figure 11 shows the MTT results from a combined exposure of HeLa and Jurkat cells in RPMI media. USEP exposure heavily impacted the viability of the Jurkat cells with little or no effect on HeLa at 150 kV/cm. While these data are not meant to be a conclusive look at the impact of USEP in heterogeneous samples, they show that large differences seen in isolated cell exposures can translate into a heterogeneous exposure system. Additionally, HeLa and Jurkat were exposed in the same medium and similar results to Figure 2 were seen suggesting that exposure of individual cultures in their respective media had little or no effect on observed differences in survival.

### Summary

This paper compared the LD_50_ for cellular death to the ED_50_ for plasma membrane disruption, using two endpoints, across various cell lines. We have shown that all cells studied externalize PS at lower doses than Pr uptake. We found that some cell lines (HeLa and CHO-K1) appear to respond only to high doses of USEP and that observed effects progressed from subtle membrane changes (PS externalization) to Pr uptake to death. In contrast, Jurkat and U937 had LD_50_ values well below the ED_50_ for Pr uptake. This finding suggests that the cell lines chosen in previous studies may have led to conclusions about specific USEP-induced cellular effects that are unlikely to be true for all cell types.

We also investigated the mechanism responsible for the observed differences in cellular survival by investigating the impact cell size, calcium concentration, and trypsinization may have on cell survival. We found that increasing calcium concentration in the external media lowered the LD_50_, and that trypsin exposure had no appreciable impact on cellular survival. By combining HeLa and Jurkat in RPMI medium, to form a heterogeneous sample, we found that we could achieve preferential kill based on the estimated dose obtained from homogenous sample exposures. These results show the potential for USEP to kill cells preferentially based on inherent susceptibilities across diverse cell lines. While this specific finding requires future work to determine the mechanism(s) that determine cellular susceptibility and proving its validity in a biologically relevant tissue, the potential of this finding for biomedical applications of USEP cannot be ignored.
